# Targeting genomic receptors in voided urine for confirmation of benign prostatic hyperplasia

**DOI:** 10.1002/bco2.362

**Published:** 2024-04-22

**Authors:** Mathew Thakur, Vivek S. Tomar, Emma Dale, Leonard G. Gomella, Charalambos Solomides, Oleksandr Kolesnikov, Scott W. Keith, Hector T. Navarro, Olivia Dahlgren, Michael Chaga, Edouard J. Trabulsi

**Affiliations:** ^1^ Department of Radiology, Urology, Radiation Oncology and S. Kimmel Cancer Center Thomas Jefferson University Philadelphia Pennsylvania USA; ^2^ Department of Radiology Thomas Jefferson University Philadelphia Pennsylvania USA; ^3^ Department of Urology and S. Kimmel Cancer Center Thomas Jefferson University Philadelphia Pennsylvania USA; ^4^ Department of Pathology and Genomic Medicine Thomas Jefferson University Philadelphia Pennsylvania USA; ^5^ Division of Biostatistics and S. Kimmel Cancer Center Thomas Jefferson University Philadelphia Pennsylvania USA; ^6^ Department of Urology Thomas Jefferson University Philadelphia Pennsylvania USA; ^7^ Department of Urology Jefferson Einstein Medical Center and Thomas Jefferson University Philadelphia Pennsylvania USA

**Keywords:** BPH diagnosis, liquid biopsy and non‐invasive BPH diagnosis, voided urine assay, VPAC receptor assay

## Abstract

**Objectives:**

The objective of this study is to validate a hypothesis that a non‐invasive optical imaging assay targeting genomic VPAC receptors on malignant cells shed in voided urine will represent either benign prostatic hyperplasia (BPH) or prostatic cancer (PCa). Risk for BPH in men 50–70 years old is 50–70% and PCa is 17%. BPH and PCa can coexist in 20% of men with BPH. Most commonly practiced methods to diagnose BPH do not distinguish BPH from PCa.

**Patients (or Materials) and Methods:**

Males with BPH (*N* = 97, 60.8 ± 6.3 years, prostate‐specific antigen 0.7 ± 0.4 ng/mL) and without oncologic disease (*N* = 35, 63.4 ± 5.8 years, prostate‐specific antigen < 1.5 ng/mL) signed informed consent form and provided voided urine. Urine was cytocentrifuged, cells collected on glass slide, fixed, treated with VPAC specific fluorophore TP4303 (Kd 3.1 × 10^−8^M), washed, incubated with DAPI and observed using a fluorescence microscope. Cells with no VPAC did not fluoresce (BPH) and those with VPAC had red‐orange fluorescence (PCa). Real‐time polymerase chain reaction analyses for VPAC and NKX3.1 assay for cell origin were performed.

**Results:**

Eighty‐seven subjects were negative for VPAC expression. Positive VPAC expression was noted in 10 subjects. Patient chart review for clinical data on these 10 VPAC positive subjects showed five had nephrolithiasis, three had renal cysts, one had prostatitis and one was being treated with finasteride. Real‐time polymerase chain reaction analysis‐VPAC expressions for 7 normal and 12 BPH subjects were 1.31 ± 1.26 and 0.94 ± 0.89, respectively (*P* = 0.46). NKX3.1 showed cells of prostate origin for finasteride‐treated patient. Specificity for VPAC urine assay for excluding prostate cancer in this BPH cohort was 88.5%, positive predictive value 0.00% and negative predictive value 100%.

**Conclusion:**

VPAC assay may contribute extensively for BPH diagnosis and warrant continued investigation.

## 1 INTRODUCTION

Benign prostatic hyperplasia (BPH) is a ubiquitous condition that inflicts millions of males worldwide. BPH has been diagnosed histologically in nearly 50% of men 50–60 years of age and nearly 70% of men 60–70 years of age.^1^ The risk of having prostate cancer (PCa) in this age group, worldwide, is 17%.^2^ BPH and PCa coexist in 20% of men older than 50, confounding early diagnosis of PCa.^3^ Combined, these conditions induce considerable morbidity and mortality in men throughout the globe. As life expectancy rates continue to increase globally, the risk of having BPH and PCa separately or in combination will only continue to rise. This will also present a challenge to distinguish BPH from PCa, in a reliable, and preferably, by non‐invasive diagnosis, as well as, in successful management of patients with these conditions.

Although BPH and PCa are distinctly different diseases in their genesis and biochemistry, they share certain common parameters such as androgen dependence and inflammatory components. Although much more is yet to be established, much is known about the two conditions that allow different pathways for their management. Because BPH shares certain lower urinary tract symptoms, not only those for PCa but also for bladder cancer, nephrolithiasis and overactive bladder, its early and accurate diagnosis is imperative.^4^

Digital rectal examination (DRE), urinalysis and blood tests for prostate‐specific antigen (PSA) are often performed to evaluate these conditions. However, they do not reliably distinguish BPH from other conditions including, and more importantly, that of PCa. Other tests such as urine flow studies, residual urine measurement, cystoscopy, ultrasound and prostate multiparametric magnetic resonance imaging (MRI) are used variably in the evaluation of BPH but they also do not distinguish BPH from PCa with high specificity. A benign enlarged prostate alone can elevate PSA blood levels; this PSA elevation raises concern for the presence of PCa. BPH is characterized by proliferation of normal stromal and epithelial cells surrounding the urethra. These cells histologically appear normal and are genomically distinct from those of PCa. A variety of urinary assays have been specifically developed that assess the relative risk of discovering PCa based on elevated PSA blood levels. An overview of biomarkers in the diagnosis and management of PCa is recently published.^5^

We have developed a simple, non‐invasive assay in which we target genomic cell surface receptors, VPAC, that are expressed in high density on malignant PCa cells shed in voided urine. Epithelial cells, also shed in voided urine in a large number, are not malignant and express relatively few VPAC receptors.^6^ Because malignant cells (MC) are shed only from tumours, patients with BPH are not expected to shed MC in their urine. The assay, therefore, provides a unique and potentially low cost opportunity to clearly define the two conditions, BPH or PCa.

For the assay, voided urine is collected using IRB approved protocol without performing DREs. Urine is then cytocentrifuged, cells collected on a glass slide, fixed and incubated with 0.5 μg of the VPAC specific biomolecule developed in our laboratory (Kd 3.1 × 10^−8^M).^7^, ^8^, ^9^, ^10^, ^11^, ^12^, ^13^ To the biomolecule is chemically attached a near infrared fluorophore and named TP 4303. When added on to the cells collected from voided urine, on a glass slide, it binds tightly to the VPAC receptors on the cells and provides orange‐red fluorescence around the MC readily visualized under a fluorescence microscope. Cells shed in urine from BPH patients are not malignant. Therefore, in the absence of VPAC receptors, they do not have orange‐red fluorescence. This virtue allows us to distinguish BPH from PCa with a high degree of confidence.

VPAC, combined for vasoactive intestinal peptide and pituitary adenylate cyclase activating peptide, encodes a cell surface G protein receptor. These receptors are expressed on many types of MC in high density on the onset of oncogenesis^6^ and provide unique target for diagnostic and therapeutic applications of many oncologic diseases.^8^, ^9^, ^10^, ^11^, ^12^, ^13^

The goal of this IRB approved (20G.196) investigation was to validate the hypothesis that this, simple, non‐invasive voided urine assay, will demonstrate the relative absence of VPAC receptors on cells, shed in voided urine of symptomatic BPH patients, thereby represent BPH and confirm the absence of PCa. The VPAC assay was also performed on urine collected from normal healthy volunteer men aged 50–70 with PSA < 1.5 ng/mL, serving as normal controls collected from subjects presenting to the urology office for other non‐malignant conditions, including nephrolithiasis and erectile dysfunction. Additionally, real‐time polymerase chain reaction analysis (RT‐PCR) and NKX3.1 assay were also performed using the cells shed in urine, which confirmed the presence or the absence of VPAC receptors and the cell origin of the cells shed in urine.

## 2 PATIENTS (OR MATERIAL) AND METHODS

Subjects aged 50–70 years (60.8 ± 6.3 years) (*n* = 97) with PSA < 1.5 ng/mL (0.7 ± 0.4) and clinically diagnosed with BPH were enrolled in this study and provided 10–50 mL of voided urine. BPH was diagnosed clinically by enlarged prostate size estimates on DRE or imaging and/or by clinical symptom presentation consistent with BPH. Additionally, 35 age matched subjects (63.4 ± 5.8 years) with PSA < 1.5 ng/mL (0.7 ± 0.4) and having no history or suspicion of any oncologic disease, BPH or lower urinary tract symptoms were also enrolled as normal controls and provided voided urine.

Digital optical fluorescent microscopy imaging with VPAC was performed on all urine samples as previously described in Supporting Information S1. RT‐PCR was performed to calculate relative VPAC expression levels in a subset of urine samples. These data were then corroborated with the results of the VPAC urine assay to provide confirmation of the urine analysis results. The assay was performed on only those urine samples from BPH and normal subjects, who provided a minimum 25 mL of urine. This volume was required to prepare slides for microscopic analysis and, additionally, be able to extract 15–20 ng of RNA required to perform the RT‐PCR assay (Supporting Information S1).

### 2.1 NKX3.1 as a marker for cells of prostatic origin

NKX3.1 testing was performed in our immunohistochemistry laboratory to determine the prostatic origin of cells, in a subset of urinary samples. NKX3.1 is a prostatic tumour suppressor gene located on chromosome 8P21.2 which provides a reliable assay to identify cells of prostatic origin^14^ (Supporting Information S1).

### 2.2 Statistical methods

Statistical analysis was performed to calculate specificity, positive predictive value and negative predictive value (NPV) among the BPH patients. These statistics were accompanied by Clopper–Pearson exact 95% confidence intervals (CIs). We could not calculate sensitivity because subjects with known PCa were not included as per the design of this study. Mean VPAC expression was compared between groups using two‐sided *t*‐tests.

## 3 RESULTS

**FIGURE 1:**
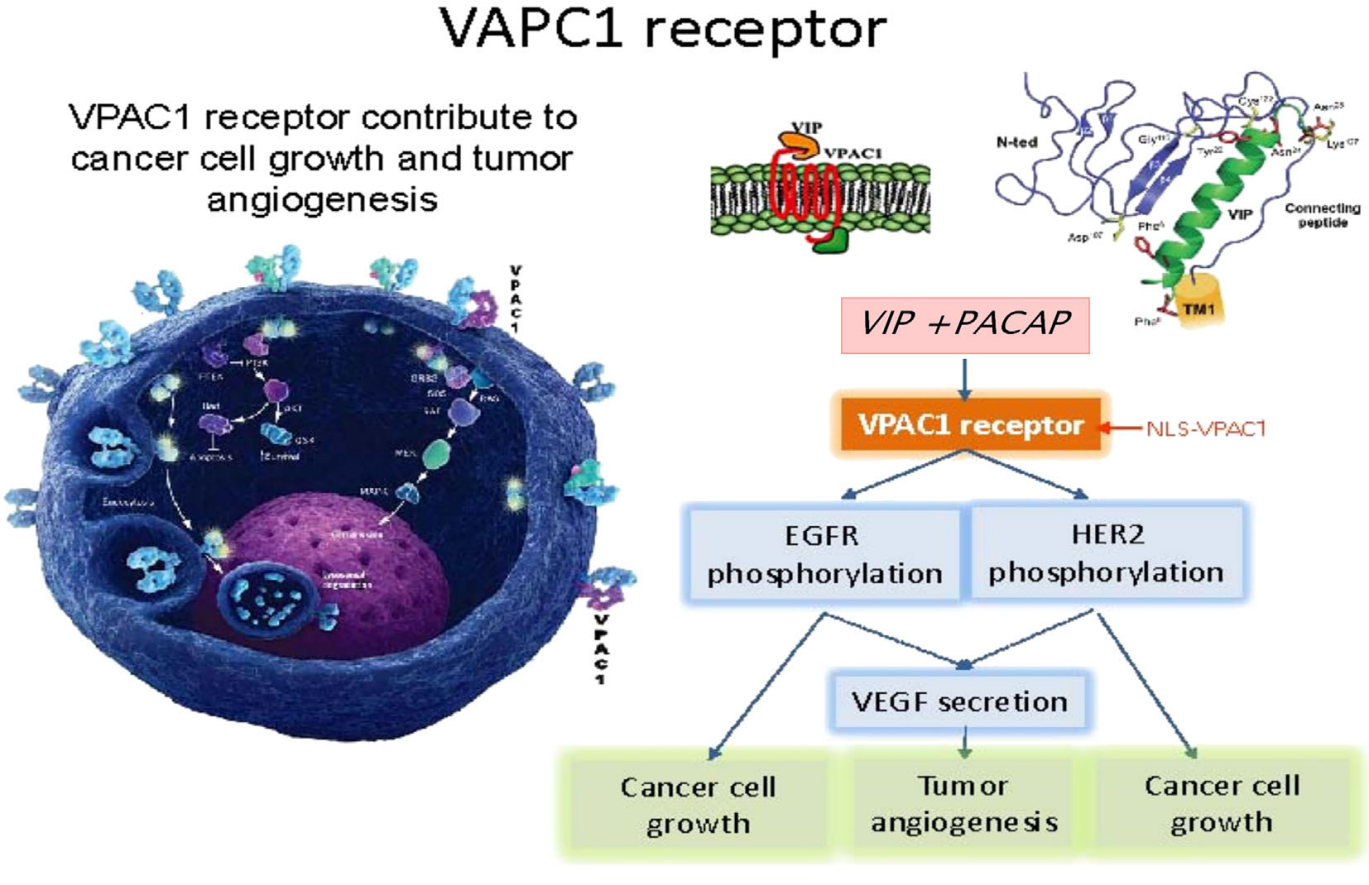
VPAC receptors have been cloned and their structure and molecular pharmacology is well defined. VPAC receptors are expressed on surface of malignant cells in high density to which TP4303 binds with high affinity leading to the identification of malignant cells or normal epithelial cells. PACAP, pituitary adenylate cyclase activating peptide; VIP, vasoactive intestinal peptide.^15^

The structure of VPAC and its role in detection of MC are outlined in Figure 1. Our long‐term goal of this ongoing investigation was to validate the hypothesis that targeting VPAC receptors expressed on cells, shed in voided urine, can diagnose BPH with high specificity and high NPV and discriminate these patients from men with PCa. Figure S1 represents the supporting data and outlines the simple procedure developed in our laboratory.

From September 2021 through July 2023, 97 male subjects (60.8 ± 6.3 years, PSA < 1.5 ng/mL, averaging 0.7 ± 0.4) who presented to the Jefferson Department of Urology office in Center City, Philadelphia, with clinically diagnosed BPH provided a voided urine sample. The urine volume provided varied from approximately 20 to 70 mL. Although the optical imaging test was performed on all samples, the RT‐PCR (12 of 97) and NKX3.1 (18 of 97) tests were performed in the subset of subjects when sufficient urine volume (>20 mL) was available. In addition, 35 urine samples were also collected from age matched male volunteers (63.4 ± 5.8 years, PSA < 1.5 ng/mL, averaging 0.7 ± 0.4) who did not have any known urothelial abnormality. When adequate urine volume was available, RT‐PCR was performed on 7 of the 35 samples collected from normal volunteers.

**FIGURE 2:**
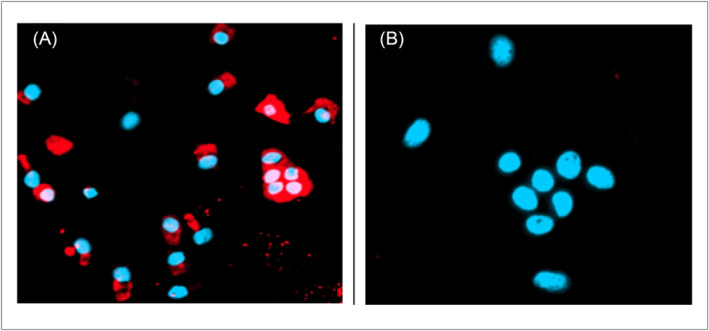
(A) Normal cells and several malignant cells on the surface of which are expressed VPAC receptors leading to red/orange fluorescence, the hallmark of the positive optical image. (B) Cell nucleus of several normal cells that do not express VPAC receptors. Such images are considered negative.

The optical imaging data analysis depicted that 87 of the 97 BPH subjects had negative imaging for VPAC positive shed cells (Figure 2A); the remaining 10 demonstrated positive optical imaging for VPAC positive shed urinary cells (Figure 2B). Evaluation of the clinical data on these 10 subjects showed that five of them had nephrolithiasis, three had renal cysts of various sizes, one had chronic prostatitis and one subject was on finasteride. Of the three subjects with renal cysts, one had renal transplant and another had squamous cell carcinoma of the bladder. Retrospectively, these subjects should not have been a part of this study but were chosen because their PSA was <1.5 ng/mL. NKX3.1 studies of 9 of the 10 subjects, excluding the one treated with finasteride, showed that the cells were not of prostate origin.

**FIGURE 3:**
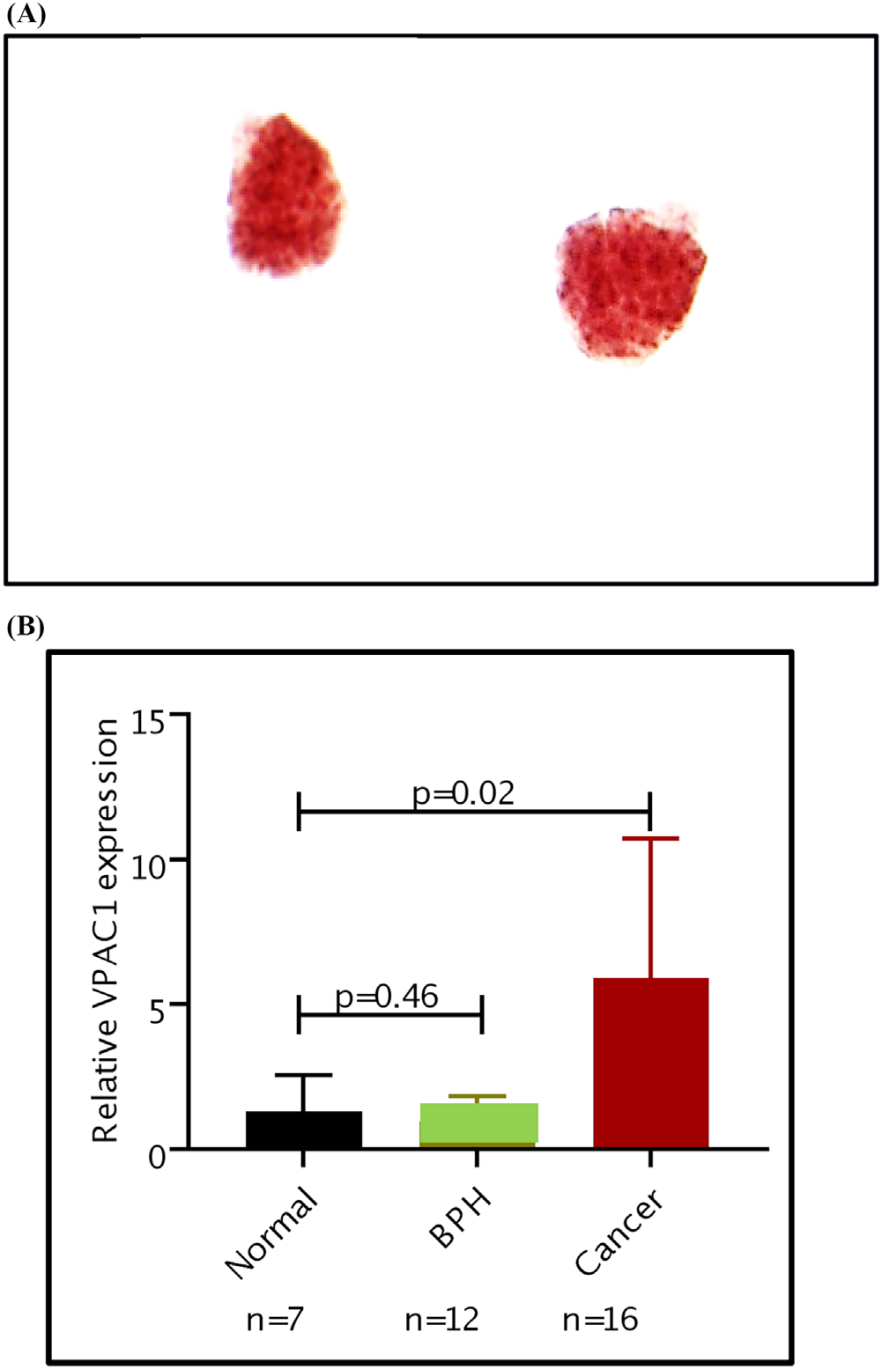
(A) NKX3.1 image of cells shed in voided urine of a benign prostatic hyperplasia (BPH) patient (prostate‐specific antigen 0.3 ng/mL) being treated with finasteride. His optical image was positive. NKX3.1 image depicts that the malignant cells were of prostate origin. (B) The histogram represents VPAC expression levels of cells shed in voided urine of normal (*N* = 7, 1.31 ± 1.26) and BPH (*N* = 12, 0.94 ± 0.89) patients. These VPAC expression levels in both types of subjects were statistically significantly different (*P* = 0.02) than those expressed in the cells of prostatic cancer patients (*N* = 16, 5.92 ± 4.8).

Among the 10 false positive BPH subjects, one was being treated with finasteride starting 9 months prior to the urine collection. His PSA was 0.4 ng/mL. Although there was no follow‐up on this subject, it is possible that this subject may have clinically unrecognized PCa. NKX3.1 study depicted that these MCs were of prostate origin (Figure 3A).

VPAC expression was determined using RT‐PCR on the urine samples of 7 normal volunteers and 12 BPH subjects who had negative optical imaging results. Their VPAC expressions averaged 1.31 ± 1.26 and 0.94 ± 0.89, respectively, which were not significantly different (*P* = 0.46). In comparison with another cohort under investigation in a separate project of subjects who are known to have PCa (*N* = 16) and positive VPAC optical imaging results, had RT‐PCR‐VPAC expression levels 5.92 ± 4.8, which was significantly higher (*P* = 0.02). These data validate the results of optical imaging (Figure 3B).

The specificity of this small study in BPH patients was 89.6% (95% CI: 81.9–94.9%), the positive predictive value was 0.0% (95% CI: 0.0–30.9%) and the NPV was 100.0% (95.9% CI: 95.9–100.0%). The false positive rate was 10.3% (95% CI: 5.0%, 18.1%).

## 4 DISCUSSION

The pharmacology and functions of VPAC receptors have been outlined in detail by Couvineau and Laburthe and Harmar et al. (Figure 1).^15^, ^16^ VPAC receptors bind with high affinity to vasoactive intestinal peptide and pituitary adenylate cyclase activating peptide. We have synthesized and evaluated a biomolecule named TP4303 that in turn binds with high affinity to VPAC.^8^, ^9^, ^10^, ^11^, ^12^, ^13^ The procedure we have developed to target VPAC receptors expressed on MC is outlined in Figure S1. This report presents the initial data obtained on this ongoing investigation in which we are set to validate the hypothesis that a simple and completely non‐invasive, VPAC targeted optical imaging assay, using voided urine, can be used to detect BPH. Although the data were limited, they demonstrated the NPV of 100% and specificity of 89.6% in confirming BPH and lack of PCa. When examining the clinical history of subjects with false positive VPAC results, the false positive results may have stemmed from the underlying patient diseases, independent of their BPH. The mechanism by which patients with nephrolithiasis (*N* = 5) and renal cysts (*N* = 3) resulted in apparent false positives is not fully understood but may be related to the genomic transformation of the cells of renal origin, collected in voided urine.^17^, ^18^ Because the normal cells shed in urine do not have VPAC receptors, they do not exhibit the VPAC‐TP4303 fluorescence. This suggests that those cells that exhibit the fluorescence must have undergone a biochemical transformation by an unknown mechanism leading to the expression of genomic receptors VPAC. The NKX3.1 study of these patients confirmed that these MC were not of the prostate origin (Figure S2). A subject known only to have chronic prostatitis was also positive by the urinary optical imaging assay. Our experimental data (Figure S3) clearly indicated that normal neutrophils do not express VPAC. Therefore, the neutrophils that must be present in the prostate due to prostatitis and shed in urine should not have expressed VPAC to lead to the false positivity. In this subject, the presence of other conditions such as a cancer of the prostate, renal or bladder was not known. The mechanistic connection of treatment with finasteride is unclear and may have caused the apparent false positive image.^19^, ^20^, ^21^ NKX3.1 assay in this patient suggested that these MCs were of prostate origin (Figure 3A). A follow‐up on this subject is necessary.

The data depict, however, that the assay may experience certain false positive results. These may include patients with bladder cancer and those with certain renal abnormalities. Despite the 10.3% rate of false positive results in this ongoing study, the specificity was 89.6% and the NPV was 100%, as compared with those of 49.6% and 82.09%, respectively, for the FDA approved select MDX test.^22^ We, therefore, trust that the results of this voided urine test are encouraging, in that this simple, non‐invasive assay can detect BPH with high degrees of confidence and is worthy of further investigations. This study does suffer from a relatively small sample size and calls for larger confirmatory studies to be performed in the future.

## 5 CONCLUSION

Our findings suggest that the VPAC targeted optical imaging assay, which uses non‐DRE voided urine, may be of a significant importance in the management of patients with BPH and warrant continued investigations.

## AUTHOR CONTRIBUTIONS

M. L. T. is the PI. He designed the experiments, supervised them, supervised data analyses, wrote the manuscript and worked with the co‐PI E. T. E. T., the co‐PI, worked with the PI, asked for consent from the patients and worked with the CRO team and the PI in finalizing the manuscript. V. S. T., E. D., H. T. N. and M. C. performed all the laboratory work and worked closely with the PI. V. S. T. also performed data analyses. L. G. is the senior consultant and took part in numerous discussions related to the project, its execution and the outcome. S. G. performed the NKX.3.1. IHC assays. S. K. performed statistical analyses. O. K. and O. D. collected urine, took care of the regulatory requirements, worked on patient demographic data and preserved patient privacy.

## CONFLICT OF INTEREST STATEMENT

Drs. Mathew Thakur and Leonard Gomella hold patents on the use of TP4303 Biomolecule stated in this manuscript. Other authors declare that they have no known competing financial interests. None have personal relationships that could have appeared to influence the work reported in this paper.

## Supporting information


**Data S1.** Supplementary Information.

**Figure S1.** The figure represents the supporting data, our underlying hypothesis (left), urine collection without DRE (top right) and the simple experimental procedure (bottom right).
**Figure S2.** NKX3.1 image of cells shed in voided urine of a patient with nephrolithiasis. Although his VPAC optical image was positive, NKX3.1 image shows that these cells were not of prostate origin.
**Figure S3.** Neutrophils were separated from venous blood of two age‐matched healthy volunteers, spread on glass slides, treated with DAPI and TP4303 as described in the VPAC optical imaging protocol. Images a. and c, were captured when cells were exposed to DAPI excitation (360 nm) and emission (460 nm) wavelength light and images b and d to the TP4303 excitation (730 nm) and emission (780 nm) wavelength light. The lack of orange/red fluorescence in b and d indicate the absence of VPAC receptors.
