# Supplementary material for: Targeting genomic receptors in voided urine for confirmation of benign prostatic hyperplasia
Source: BJUI Compass. 2024 Apr 22;5(7):675–80. doi: 10.1002/bco2.362 (PMC11250152; doi:10.1002/bco2.362)
Supplement: Supplementary file 1 — Data S1. Supplementary Information. [file BCO2-5-675-s002.docx]

**Supplement information- Research Methodology:**

**Digital optical fluorescent microscopy imaging:**

From the time of collection to the time of their laboratory processing, the urine samples were stored at 5 °C in a refrigerator or in a cold room, for up to 72 hours. Any sample with mild hematuria was subjected to hypotonic lysis. Any turbid sample was centrifuged at 750xg for 3 minutes, supernatant was carefully separated and was further centrifuged at 2000xg for 10 minutes. All but 1 ml of the urine supernatant was then removed and cells were gently re-suspended. Two hundred µl cell suspension was cytocentrifuged at 2000xg for 20 minutes. Cells on the glass slide were then fixed in 95% ethanol for 10 minutes and slides were allowed to air dry. The receptor specific biomolecule (0.5 µg) TP4303, in 150 µl of phosphate buffered saline (0.1% tween-20) was added on the slide, to cover the entire cell area and was incubated in dark for 10 minutes. The excess of TP4303 was thoroughly washed and slides were dried in a dark box with airflow, for 10 minutes. One hundred fifty µl of DAPI (4, 6-diamidino 2 phenylindole), was placed on the cells, incubated for 15 minutes, also in dark and cover slip was placed, free of any air bubbles. Slides were then examined using Zeiss Axio Observer microscope coupled with Zenn Blue microscopy software. The microscope was covered with a dark plastic box that prevented stray light exposure to TP4303. The Zenn software allowed us to determine the total number of normal epithelial cells and the VPAC expressing PCa MC present on each slide. The presence of MC depicts PCa and their absence determines BPH.

**Real-time polymerase chain reaction analysis (RT-PCR):**

For Rt-PCR studies, 15 ml to 25 ml of urine was centrifuged at 1500 rpm for 10 minutes at room temperature. An RNA minikit (Invitrogen, Carlsbad, CA) was used. After centrifugation, the cells were lysed using 300 µl of lysis buffer containing 1% ß - mercaptoethanol (Sigma, St. Louis, MO). Mixture vortexed, 450 µl 100% ethanol were added, the mixture was loaded onto a mini column (Invitrogen), centrifuged and the column washed thrice, using 700 µl buffer also provided by Invitrogen. RNase free water (20 µl) was then added onto the column and spun for 2 minutes at 12000 rpm. The RNA extracted was measured using a calibrated nanodrop spectrophotometer.

The RNA was then converted into cDNA using the high capacity RNA to DNA kit (Applied Biosciences, Beverly Hills, CA). Using nuclease free water, the cDNA was diluted to 1 ng/ µl and used to perform real-time quantitative PCR (Applied Biosystems, Quantstudio 3) which assessed the threshold cycle (CT) values for VPAC1. GAPDH (glyceraldehyde 3–phosphate dehydrogenase) was used as a control. Human primers (FAM-MGB, HS00910453) for VIPR1 gene and (FAM-MGB, HS03929097) for GAPDH obtained from Applied Biosystems were used and Tagman assay procedure was carefully followed. Delta-delta CT values were then calculated. These values determined the relative fold gene expression on cells shed in voided urine. These studies provided the biological and genomic status of the cells shed in voided urine for validation of the optical imaging data obtained on the cells shed in the same urine sample.

**NKX3.1 as a marker for cells of Prostatic Origin:**

The assay was performed on cytocentrifuged urine cells, using Roche Ventana Benchmark ULTRA staining platform. The primary NKX3.1 antibody was obtained from Biocare (PP422AA), incubated with the cells at 37 °C for 45 minutes. For secondary immunostaining, horseradish peroxidase multimer cocktail (Roche) was used. The immune complexes were visualized using Ultraview Universal DAB (diaminobenzidine tetrahydrochloride) detection kit, also obtained from Roche. The signal was amplified with rabbit antimouse IgG heavy and light chain antibody. Slides were then washed with tri-based reaction buffer (Roche) and counter stained with Hermotoxylin II (Roche). The cells of prostate origin stained dark brown.
