# Supplementary material for: Targeting genomic receptors in voided urine for confirmation of benign prostatic hyperplasia
Source: BJUI Compass. 2024 Apr 22;5(7):675–80. doi: 10.1002/bco2.362 (PMC11250152; doi:10.1002/bco2.362)
Supplement: Supplementary file 2 — Figure S1. The figure represents the supporting data, our underlying hypothesis (left), urine collection without DRE (top right) and the simple experimental procedure (bottom right). Figure S2. NKX3.1 image of cells shed in voided urine of a patient with nephrolithiasis. Although his VPAC optical image was positive, NKX3.1 image shows that these cells were not of prostate origin. Figure S3. Neutrophils were separated from venous blood of two age‐matched healthy volunteers, spread on glass slides, treated with DAPI and TP4303 as described in the VPAC optical imaging protocol. Images a. and c, were captured when cells were exposed to DAPI excitation (360 nm) and emission (460 nm) wavelength light and images b and d to the TP4303 excitation (730 nm) and emission (780 nm) wavelength light. The lack of orange/red fluorescence in b and d indicate the absence of VPAC receptors. [file BCO2-5-675-s001.pptx]

## Slide 1
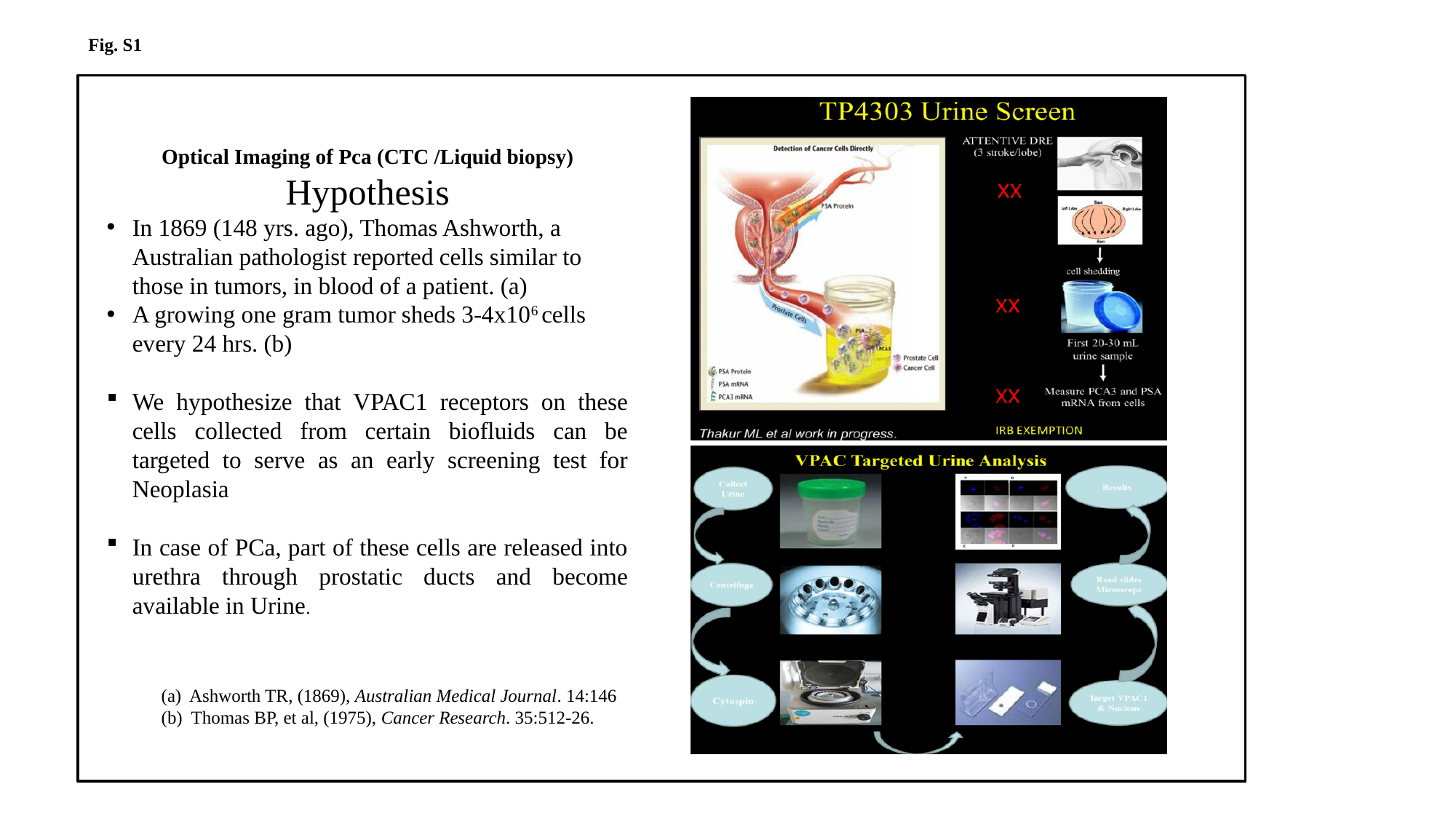

Fig. S1
Optical Imaging of Pca (CTC /Liquid biopsy)
Hypothesis
In 1869 (148 yrs. ago), Thomas Ashworth, a Australian pathologist reported cells similar to those in tumors, in blood of a patient. (a)
A growing one gram tumor sheds 3-4x10^6^ cells every 24 hrs. (b)
We hypothesize that VPAC1 receptors on these cells collected from certain biofluids can be targeted to serve as an early screening test for Neoplasia
In case of PCa, part of these cells are released into urethra through prostatic ducts and become available in Urine.
(a) Ashworth TR, (1869), Australian Medical Journal. 14:146
(b) Thomas BP, et al, (1975), Cancer Research. 35:512-26.

## Slide 2
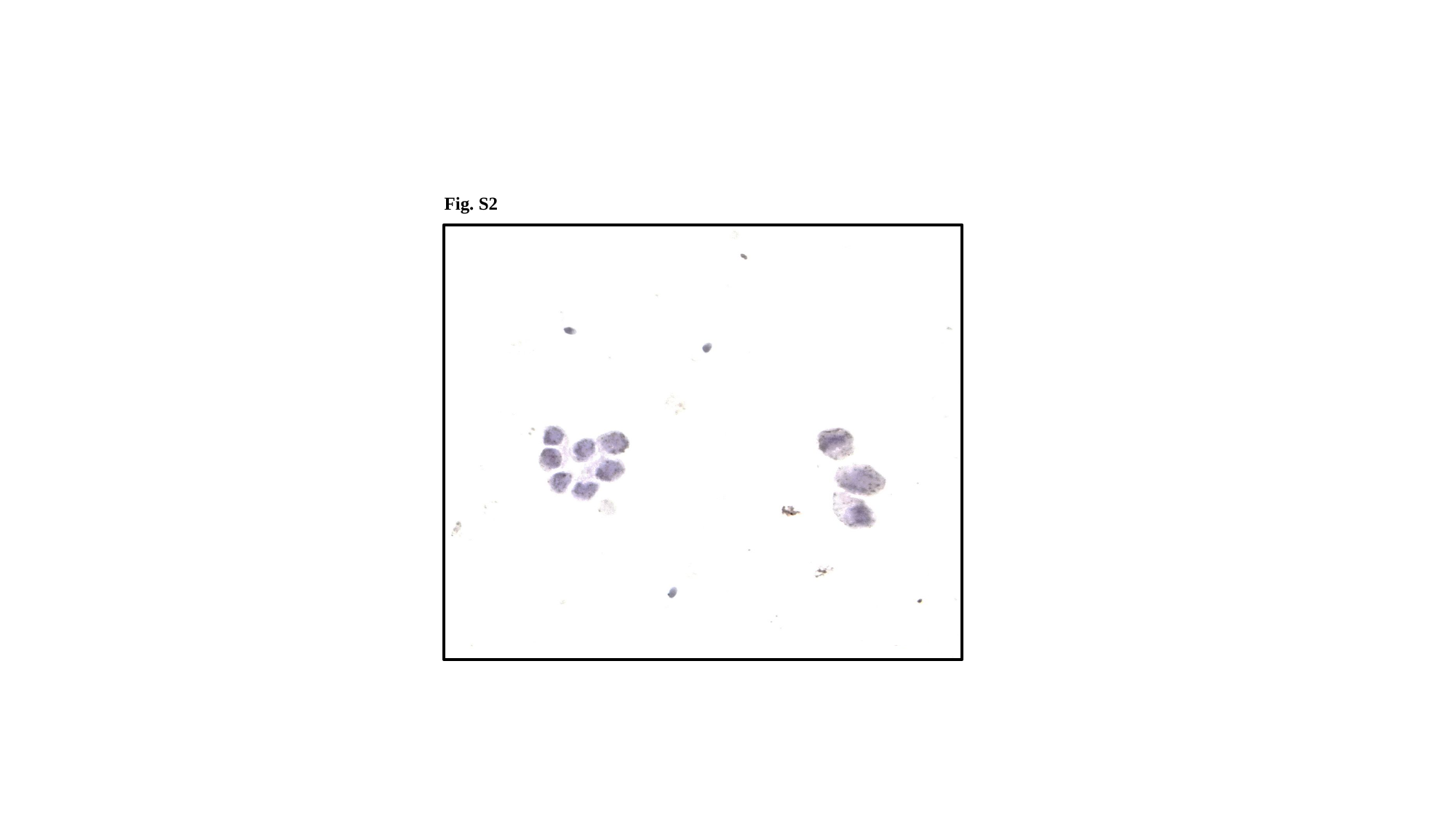

Fig. S2

## Slide 3
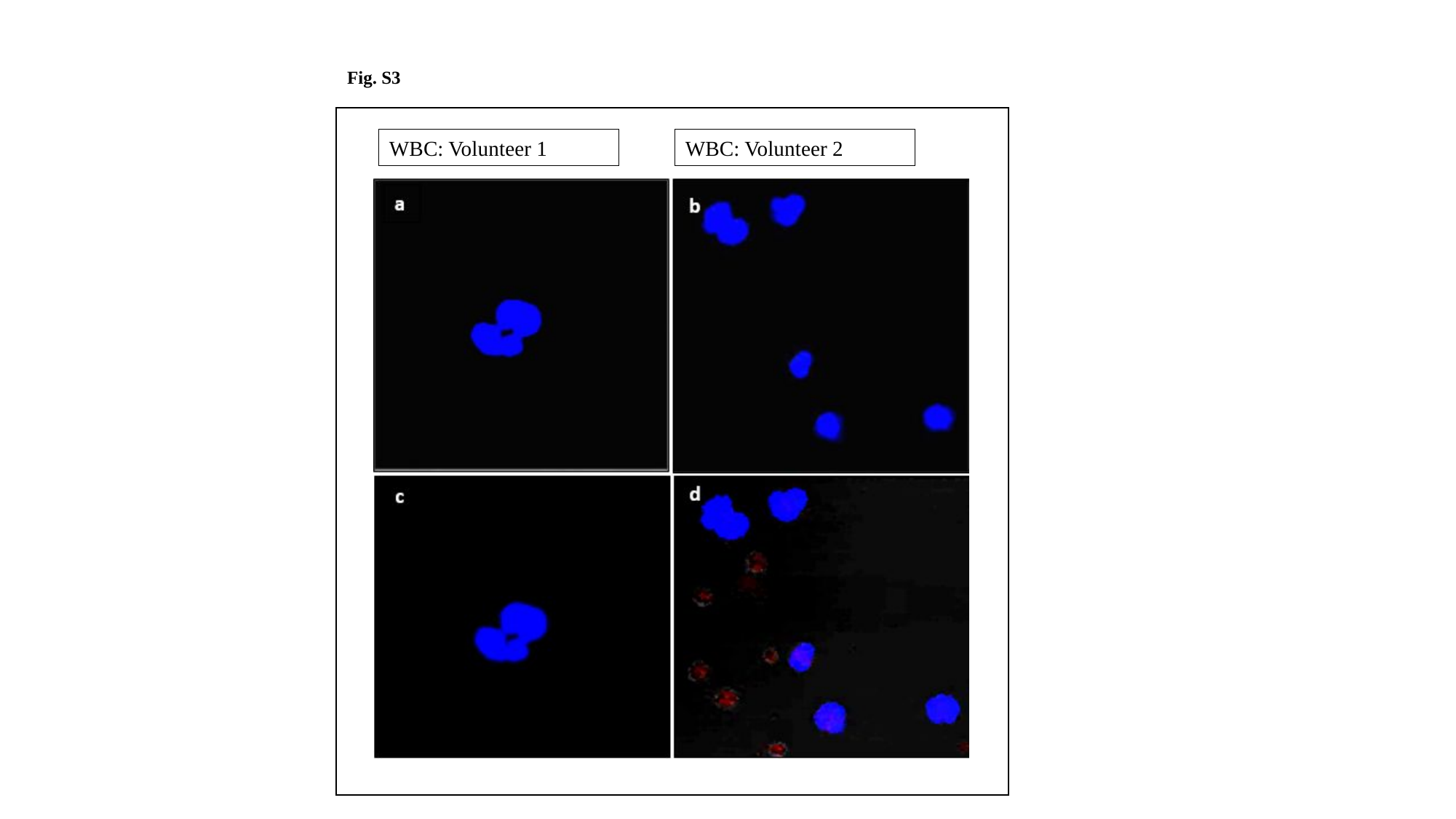

Fig. S3
WBC: Volunteer 1
WBC: Volunteer 2
b
d
b
d
